# Prevention of Progression in Myopia: A Systematic Review

**DOI:** 10.3390/diseases6040092

**Published:** 2018-09-30

**Authors:** Aldo Vagge, Lorenzo Ferro Desideri, Paolo Nucci, Massimiliano Serafino, Giuseppe Giannaccare, Carlo E. Traverso

**Affiliations:** 1 Eye Clinic of Genoa, Policlinico San Martino, Department of Neuroscience, Rehabilitation, Ophthalmology, Genetics, Maternal and Child Health (DiNOGMI), University of Genova, 16132 Genova, Italy; mc8620@mclink.it; 2 School of Medicine and Pharmacy, Department of Neurosciences, Rehabilitation, Ophthalmology, Genetics, Maternal and Child Health (DiNOGMI), University of Genoa, 16132 Genoa, Italy; lorenzoferrodes@gmail.com; 3 University Eye Clinic San Giuseppe Hospital, University of Milan, 20162 Milano, Italy; paolo.nucci@unimi.it (P.N.); massimiliano.serafino@multimedica.it (M.S.); 4 Ophthalmology Unit, Department of Experimental Diagnostic and Specialty Medicine (DIMES), University of Bologna, S. Orsola-Malpighi Teaching Hospital, 40138 Bologna, Italy; giuseppe.giannaccare@gmail.com; 5 IRCCS Ospedale Policlinico San Martino, 16132 Genova, Italy

**Keywords:** myopia, myopia prevention, atropine, ATOM, orthokeratology, spectacles

## Abstract

The prevalence of myopia has increased worldwide in recent decades and now is endemic over the entire industrial world. This increase is mainly caused by changes in lifestyle and behavior. In particular, the amount of outdoor activities and near work would display an important role in the pathogenesis of the disease. Several strategies have been reported as effective. Spectacles and contact lenses have shown only slight results in the prevention of myopia and similarly ortokerathology should not be considered as a first-line strategy, given the high risk of infectious keratitis and the relatively low compliance for the patients. Thus, to date, atropine ophthalmic drops seem to be the most effective treatment for slowing the progression of myopia, although the exact mechanism of the effect of treatment is still uncertain. In particular, low-dose atropine (0.01%) was proven to be an effective and safe treatment in the long term due to the lowest rebound effect with negligible side effects.

## 1. Introduction

Myopia, or ‘nearsightedness’, is the one of the most common refractive disease worldwide and is due to an excessive axial elongation of the eye as a major mechanism in children [1]. According to the recent report published in Nature [2], myopia is becoming an epidemic in the developed countries of East and South-East Asia, where the prevalence reaches peaks of 80–90% in children attending secondary school, aged 17–18. Concomitantly, the European Eye Epidemiology Consortium (E_3_) demonstrated in a meta-analysis of a population based, cross-sectional study that also in the Western countries the prevalence of myopia is dramatically growing, with a significant variability between age groups, resulting higher (46%) in the 25 (years-old) subgroup than the 75 (years-old) one, with only 15% people affected [3].

In a recent meta-analysis Holden et al. estimated in the world population the exponential growth of myopia and high myopia, defined as loss of 6.00 diopters (D) or more, increasing respectively from 1406 million (22.9%) and 163 million (2.7%) in 2000 to 4758 million (49%) and 938 million (9.8%) in 2050. These alarming data imply that in 2050 half of the world population may be affected by myopia and it would represent a significant social and economic burden to the global healthcare systems [4].

By contrast, a similar trend has not been described in all the countries; in a Danish cross-sectional study Jacobsen et al. reported a significant decrease in high myopia from 1882 onwards and a decreasing tendency from 1964 to 2004. More in detail, in the examined sample including 4681 male Danish conscripts, the authors found that the prevalence of myopia (SE ≤ −0.5 D) was 12.8% and high myopia (SE < −6.5 D) only 0.3% [5]. In addition, French et al. compared the prevalence of refractive errors in European Caucasian children aged 6 to 7 years and 12 to 13 years, living in Sydney, Australia and in Northern Ireland and showed a relatively low prevalence of myopia, 5% and 15.0%, respectively, in each sample [5].

Nonetheless, the risk of high myopia-related pathological complications in the macula, in peripheral retina, in the optic nerve and in the lens must not be neglected. In this regard, the Rotterdam Eye Study pointed out in a population-based cohort study the presence of bilateral visual impairment in high myopic eyes, with 39% of the patients affected by myopic maculopathy, 17% by open-angle glaucoma and 5% by cataracts [6]. In addition, it has been demonstrated that an early onset of myopia, is more dramatically related to a more severe degree of the disease during the adult age [7].

The aim of our review is analyzing all the possible behavioral, interventional and pharmacological strategies that can be adopted in the pediatric population, in order to slow the progression of myopia. The recent network meta-analysis published by Huang et al. compared the effectiveness of different interventions used to slow down the progression of myopia, including atropine and other anti-muscarinic agents, orthokeratology (OrthoK), soft bifocal contact lenses (SCLs), bifocal spectacles (PBSLs) and progressive lens spectacles (PASLs) and more outdoor activities [8].

In our review we will discuss not only the clinical efficacy of the investigated interventions, but we will also evaluate the side effects, the patient tolerability and the effective long-term advantages for the pediatric population.

## 2. Methods

In this review article a systematic computerized search of the literature was conducted from the inception until December 2017. All English-language articles dealing with the topic of myopia control therapies were searched in the electronic databases PubMed, MEDLINE and the Cochrane Collaborations and selected by the authors. The following keywords and MeSH terms were used: “Myopia” alone or in combination with “control”, “progression”, “pediatrics”, “prevention”, “atropine”, “orthokeratology”, “contact lenses”, “spectacles”, “outdoor activities”, “near work”, “epidemiology”, “genetics”, “pathogenesis”, “dopamine”. All the pertinent articles were thoroughly assessed and their reference lists were evaluated in order to identify any other study that could have been included in this review article.

The reviewers were not blinded to the names of the investigators or the sources of publication. The eligibility of the studies was first assessed on titles and abstracts. Full manuscripts were achieved for all chosen studies and decision for final inclusion was made after thorough examination of the papers.

## 3. Etiopathogenesis

Myopia is a multifactorial disease caused by the deep interaction between genetic factors, including parental myopia and ethnicity, and environmental factors.

### 3.1. Genetic Background

It has been recognized the positive association between parental myopia and the risk for the child of developing myopia [9,10]. Ip et al. showed the significant influence of the parental myopia and of the ethnicity on spherical equivalent refraction and axial length on a sample of 12-year-old Australian children [10]. While common myopia is generally transmitted as a complex trait, high myopia can be transmitted either as a complex trait or a Mendelian trait, including autosomal dominant (AD), autosomal recessive (AR), and X-linked recessive (XL) inheritance. In this regard, linkage analysis has allowed us to identify 18 myopia and high myopia loci, like MYP2, MYP3 or MYP5, giving us the opportunity to screen possible candidate genes responsible for the disease [11]. In order to better understand the heritability of myopia and to analyze myopic traits, several studies on twins have been conducted over the years in monozygotic twins (MZ) [12,13,14]. In this regard, the Twin Eye Study demonstrated that the heritability of myopia in MZ twins was 90% (95% CI, 81–95%) [15]. A recent cohort study, the Guangzhou Twin Eye Study identified first the important role played by the peripheral refraction in myopia progression, suggesting also its significant genetic contribution (heritability 55–84%) [16]. Moreover, experimental myopia studies have allowed us to better characterize the genes involved in the pathogenesis of myopia. These genes have in common that they can regulate ocular growth, they can also be modulated in induced-myopia models, and, nonetheless, they are ubiquitously expressed in the sclera, choroid and retina. Among them, TGF-beta/BMPs pathway seems to display an important role, since animal models have shown that the reduced expression of TGF-beta isoforms in the sclera was associated with a decreased synthesis of collagen and therefore a more consistent predisposition to pathological axial elongation [11]. However, genome-wide association studies (GWAS) have assessed that the susceptible loci causing refractive error have only a marginal role in the risk of developing myopia (0.5–2.9%) [17]. This would suggest a stronger relevance displayed by other non-genomic factors, such as epigenetic changes and the environment. In fact, in a prospective cohort study including a population of 5,257 participants, Verhoeven et al., comparing subjects with the same degree of genetic risk, highlighted how environmental factors such as higher levels of education were more significantly associated with the onset of myopia rather than lower levels of education [18]. Hence, we should consider myopia as a more complex mosaic, where the gene-environment interaction is crucial in order to develop the disease.

### 3.2. Physiopathology and Biological Mechanisms of Myopia

Since 1977 when Wiesel and Raviola accidentally discovered in animal models that visual form deprivation was implied in genesis of myopic axial elongation, the local control of retina on the eye growth was postulated [19]. Several animal models have shown the response of axial eye growth in order to compensate an imposed defocus [20,21,22]. In non-human primate models, it has been demonstrated that manipulating the peripheral retinal defocus with bifocal contact lenses can modify the eye growth and the refractive status. More in particular, axial myopia can be produced by imposing a peripheral hyperopic defocus [23,24]. In another study on marmosets, Benavente-Perez et al. suggested that imposing a positive defocus on retina, could be considered an effective strategy in order to slow myopia progression [25]. Additionally, it was found that this response could be elicited locally in the eye globe if the visual deprivation was specifically directed to certain retinal areas.

Thus, retina is involved in the modulation of the eye axial length when a sign of defocus is detected. More surprisingly, the underlying choroid can modify its thickness to move the retina closer to the focal plane [26].

However, applying on human models an imposed defocus would cause smaller modifications in eye axial length than animal experiments. In this regard, the COMET and STAMP clinical trials observed in children treated with progressive addition lenses a smaller modification in the refraction in proportion to the degree of refractive error imposed. Thus, they hypothesized that a more gradual accommodation, induced in children with progressive addition lenses, would reduce the retinal peripheral defocus and, therefore, the stimulus for the eye growth. Anyway, further clinical studies are needed to better establish the role of accommodation in relenting the retinal error signal and the consequent inhibition of eye growth in children.

Many bio-molecular pathways have been investigated in the pathogenesis of myopia. Although the role of cholinergic, nitric oxide (NO) and, more recently, insulin pathways have been studied, the importance of dopamine (DA) remains crucial in the development of experimental myopia [27,28,29].

Reduced levels of DA in chicks related to visual deprivation were first demonstrated by Stone et al. in 1990 [30]. Several other studies confirmed that chicks treated with negative lenses had decreased values of DA primary metabolite, the 3,4-Dihydroxyphenylacetic acid (DOPAC), in the vitreous body [31,32]. These findings would suggest an inverse relationship between dopamine levels and myopic eye growth. Based on this assumption, dopaminergic drugs were experimented as anti-myopic agents in animal models. Several other studies confirmed that chicks treated with negative lenses had decreased values of 3,4-Dihydroxyphenylacetic acid (DOPAC), the primary metabolite of DA, in the vitreous body [33,34]. DA exerts its effect through both D1-like receptors (D1 and D5 receptors), which are located on bipolar, apocrine and ganglion cells, and through D2-like receptors (D2, D3, D4), situated on retinal pigment epithelium cells (RPE) and neuroepithelial cells [35]. Recent findings have extended the traditional view where D2-like receptors displayed the main role in the development of myopia [36] and supported the evidence of a combined action between D1-like receptors and D2-like receptors [37]. Furthermore, researchers have focused great attention on the role of RPE in modulating eye growth. The RPE has been shown to be responsible for the release of growth factors regulating scleral remodeling, in response to experimental form deprivation myopia (FDM) and flickering light induced myopia (FLM) [38,39]. The recent study published by Luo et al. revealed paradoxical increased levels of dopamine in the RPE of guinea pigs with FLM [39]. This evidence would suggest the presence of different retinal pathways causing FDM and FLM, but the RPE would represent the final common modulator in the myopic scleral remodeling. Other studies have investigated the possible role of other molecules involved in myopia progression. The administration of 7-methylxanthine in young rabbits has shown to the administration of 7-methylxanthine in young rabbits has shown to reinforce the posterior sclera increasing its collagen fibrils content [40]. In addition, in guinea pigs 7-mx has been prove to slow down the amount of eye elongation induced by form deprivation, preventing the thinning of posterior sclera Thus, given the results in animal models, methylxanthines have been investigated as possible treatment options in the prevention of myopia [41].

On the other hand, also the systemic administration of melatonin in chicks has been proven to be associated with choroidal thinning [42]. A recent study performed in humans has shown that myopic subjects have higher serum levels of melatonin combined with lower serum dopamine in comparison with non-myopic subjects. These findings would reinforce the important role displayed in the development of myopia by light exposure and circadian rhythm, which are strongly connected to melatonin metabolism [43]. In this regard, it is known the protective effect of light exposure against the development of deprivation myopia. Animal models like chicks and monkeys have revealed that the onset of experimental myopia can be limited by the daily exposure to bright light (15,000–30,000 lux) [44,45]. Moreover, a proportional effect of light intensity in diminishing the refractive error has been demonstrated in chicks [46]. Hence, light exposure has proven to display an important role in the prevention of myopia, due to its modulator effect on dopamine and other biomolecules release [47]. Thus, in the near future, other studies are necessary to define the complex role played by DA and other possible neurohormones involved in the onset of myopia.

## 4. The Role of the Environment

### 4.1. Outdoor Activities

The evidence of a protective effect of the outdoor activities have been investigated in many epidemiological studies. The Guangzhou randomized trial evaluated over a period of 3 years the efficacy of a daily additional outdoor activity in a sample of 952 children of 6–7 years old, comparing the interventional group with a control group of 951 children of the same age with the usual pattern of outdoor activities [48]. The interventional group showed a reduction in the spherical equivalent refraction (myopic shift) compared to the control group (mean of −1.42 D vs. −1.59 D, respectively; difference of 0.17 D [95% CI, 0.01 D to 0.33 D]; *p* = 0.04). In this study the increase of outdoor activities entailed a relative reduction of 23% of the incidence of myopia in the interventional group. These findings concurred with the prospective, interventional study published in by Wu et al., where a recess outside the classroom program (ROC) was performed in a sample of 571 children in Taiwan [49]. It was demonstrated that more outdoor activities led to a reduction of the 8.4% in the onset of myopia in the ROC group compared to the control group after 1 year (8.41% vs. 17.65%; *p* < 0.001). The “Anyang Childhood Eye Study “revealed in some population of 2276 Chinese children aged 10–15 the significant association between outdoor activities and a slower axial elongation rate in children not myopic at baseline (high versus low tertile, −0.036 mm/year; *p* = 0.009) [50]. On the contrary, children already myopic at baseline did not show a significant effect of time spent outdoor on the reduction of axial elongation (high versus low tertile −0.005 mm/year; *p* = 0.595).

Also, the recent “Handan offspring myopia study” reported a significant inverse relationship between outdoor activities and incidence of myopia in rural children in China, however the size of effect was small (OR, 95% CI: 0.82, 0.70–0.96) [51].

All these studies demonstrate that outdoor activities have a more important impact on reducing the onset of myopia, rather than slowing down the progression of the disease. In fact, the recently published meta-analysis by Xiong et al. reported that more time spent outdoor would display a protective effect for both the incidence (clinical trials: risk ratio (RR) = 0.536, 95% confidence interval (CI) = 0.338 to 0.850; longitudinal cohort studies: RR = 0.574, 95% CI = 0.395 to 0.834) and the prevalence of the disease (cross-sectional studies: OR = 0.964, 95% CI = 0.945 to 0.982), but in dose-response analysis was not found a significant association between more outdoor activities and myopia progression (R^2^ = 0.00064) [52].

Another important element for the development of myopia could be attributed to the residential area where children live. A cross-sectional study of school children in Indonesia revealed that the prevalence of uncorrected refractive error was 10.1%, 12.3%, 3.8%, and 1%, respectively among urban, suburban, exurban and rural area [53]. Thus, the higher prevalence among urban and suburban area could be explained with a lack of outdoor activities combined with a more intensive near work due to the higher education in comparison with rural areas. Moreover, according to the study published by Read et al., the daily light exposure would exhibit a more important role in the development of myopia in Australian children, rather than the amount of physical activity [54].

In closing, the biological mechanism explaining how time spent outdoor reduces the onset of myopia still needs to be better clarified. Studies in animal models reinforced the role of DA in association with light exposure, already hypothesized by Rose et al. [55]. In fact, Ashby et al. referred in chicks exposed to high luminance levels the failure to retard myopia development, when injected with spiperone (500 μM), a D2-dopaminergic antagonist [28]. Further studies must be performed in the future, in order to elucidate the complex relation between dopamine levels, sun light exposure and outdoor activities.

### 4.2. Near Work Activity

Near work activity has been investigated in many studies as an independent risk factor for myopia, however, unlike outdoor activities, a consistent evidence must still be provided. A Singapore myopia study, reported that teenagers spending more than 20.5 h a week reading and writing were significantly more likely to develop myopia (odds ratio 1.12, 95% CI 1.04–1.20, *p* = 0.003) [56].

The Sydney myopia study analyzed in a population of 12-years old Australian children the impact of close reading distance (<30 cm) and continuous reading (>30 min) on the spherical equivalent refraction (SER), finding respectively a 2.5 times and 1.5 more probability to develop myopia compared to the control group. (*p* = 0.02 and *p* = 0.0003) [10]. Despite of the relevance of these independent variables, the overall time spent on near work activities was not significant in multivariate analyses for myopia (SER ≤ −0.50 D). Another 3-year follow-up cohort study, the Beijing Myopia Progression Study, revealed a positive association between myopia and near work only in the group of students with a greater near work load at the baseline (hazard ratio, 95% confidence interval: 5.19, 1.49–18.13) [57]. By contrast, other studies reported no significant correlation between near work activities and refractive error onset [49]. For example, Rose at al. did not find any relevant changes in myopia prevalence in 6-years old children despite of low (0–2 h), moderate (1.6–3.1 h), high (>3.0 h) daily amount of near work activities [55].

The recent systematic review and meta-analysis published by Huang et al. included 12 cohort studies and 15 cross-sectional studies on the association between myopia and near work in children [58]. The results reported that more near work activities were related with higher odds of myopia (odds ratio [OR] = 1.14; 95% confidence interval [CI] = 1.08–1.20) and that the odds of myopia increased by 2% (OR:1.02; 95% CI = 1.01–1.03) for every one diopter-hour more of weekly near work.

More in general, several studies have demonstrated a strong positive association between higher tiers of education and the prevalence of myopia; these results could be explained by different factors, including both the increase of near work activity and, nonetheless, a concomitant reduction of outdoor activities in more educated subjects [12,59,60].

Thus, this evidence would confirm the multifactorial pathogenesis of myopia, in which near work activity constitutes an important independent risk factor.

## 5. Treatments

Clinical studies investigating the efficacy of treatments for myopia progression are summarized in Table 1.

### 5.1. Biofeedback Visual Training

From Bates’ theory in the 1920s it was hypothesized that an overwork of extra-ocular muscles could cause changes in the accommodation. In particular, biofeedback visual training would modify the action of the autonomic nervous system on the accommodative process [69]. Rupolo et al evaluated in a non-randomized prospective study including 33 female students the efficacy of visual training through the use of an acoustic biofeedback technique. After 12 months, no significant differences were shown between the treated and the control groups [70]. Analogously, previous non-randomized clinical studies reported no efficacy of this treatment modality [71,72,73]. More recently, a case-control study evaluated the efficacy of Chinese eye exercises in controlling myopia in a sample of 261 children (mean age 12.7 ± 0.5 years) and overall found no significant association between eye exercises and the risk of developing myopia (OR = 0.73, 95% CI: 0.24–2.21), nor myopia progression (OR = 0.79, 95% CI: 0.41–1.53) over a 2-year-follow-up period [74].

In conclusion, no consistent evidence has been shown that biofeedback visual training could be effective in slowing down myopia progression.

### 5.2. Spectacles and Contact Lenses

Progressive additional lens spectacles and bifocal spectacles.

In the daily clinical practice single-vision spectacles lenses (SVSLs) and contact lenses are commonly prescribed to children for the correction of myopia. However, progressive additional lenses (PALs) and bifocal lenses spectacles are occasionally prescribed for the purpose of slowing down the refractive disease. The underlying mechanism behind PALs treatment must be better understood, but several studies would suggest an effect on the reduction of the retinal hyperopic blur by reducing the accommodative lag during near work [75,76]. By contrast, a 3-year Finnish RCT enrolling 240 schoolchildren aged 9 to 11 reported that myopia progression was more associated with the amount of near work and diminishing the accommodative effort by the use of bifocal or reading spectacles was ineffective [77].

The randomized Correction of Myopia Evaluation Trial (COMET) enrolled 469 children aged 9 and randomly assigned to the PALs group, with a +2.00 addition, or to SVSLs group, with prescription of the common spectacles used for myopia. The progression of myopia was measured by autorefraction after cyclopegia over a period of 3 year-follow up, finding a statistically positive gain of only 0.2 D and 0.11 m in the PALs group [78,79]. Therefore, despite of the statistical significance, the clinical effect was small. Relying on these data, Gwiazda et al. highlighted in subgroup analyses a greater effect on children with larger accommodative lags (>0.43 D for a 33 cm target) combined with near esophoria (PAL-SVL progression = −1.08 D − [−1.72 D] = 0.64 ± 0.21 D) [68].

The following COMET 2 Study, another double- masked multicenter randomized trial, elected for PALs treatment only myopic children with near esophoria and at least 1.00 D of accommodative lag, defined as an accommodative response less than 2.50 D for a 3.00-D demand. Considering a 3 year-follow up, they found a gain of the spherical equivalent refraction (SER) of only 0.28 D for the PALs group, in comparison with the SVSLs one [80]. The reduction of myopia progression using PALs resulted not clinically significant again.

Bernstein et al. randomized 85 children with high accommodative lag to wear either PALs or SVSLs the first year, while all the children wore SVSLs the second year. The PALs treatment effect was a 0.18 D gain after one year wearing PALs (*p* = 0.01), but the second year no difference was shown in myopia progression between the groups [81]. In addition to the already known weak clinical effect [82], they showed no association between accommodative lag and myopia progression, suggesting a clinical irrelevance of PALS in decreasing the foveal blur during near work.

The recent trial published by Hasebe et al. aimed to compare the clinical efficacy of newly designed positively aspherized progressive addition lenses (PA-PALs) with the traditional PALs. PA-PALs, working on the reduction of both lag of accommodation and peripheral lag in the peripheral retina, caused a 0.27 ± 0.11 retardation of myopia progression during a 2 year-period, in agreement with PALs percentage efficacy ranges [83].

On the contrary, Cheng et al. evaluated the effect of bifocal spectacles (BSLs) and prismatic bifocal spectacles on the reduction of myopia progression in a preselected sample of Chinese-Canadian children with high rate myopia (at least 0.50 D loss/year). Across the 3 year-follow-up period, this randomized, clinical trial reported a significant reduction in myopia progression of −0.81 D (*p* < 0.001) and −1.05 D (*p* < 0.001) in the bifocal lens and prismatic bifocal lens groups, respectively, in comparison with the single-vision lens groups. Furthermore, both bifocal groups resulted more efficacious during the first year of treatment and, overall, no significant difference was shown between the bifocal and the prismatic bifocal groups [84]. However, the positive results of this study could have been influenced by a population bias; in fact, Leung et Brown reported better visual outcomes wearing progressive lenses in myopic children with high myopia progression [85]. In fact, in contrast with multifocal lenses, the design of bifocal lenses would allow the segment line to work as a reminder to the child to deliberately use the reading portion of the bifocal lenses while performing near work and take advantage of a greater visual field [86].

In conclusion, as the recent meta-analysis published by Huang reported, the clinical effect of both PALs and BSLs resulted modest in comparison with SVSLs or placebo with, respectively, a mean percentage difference in refractive changes of 0.17 (0.00–0.034 credible interval [CrI]) and 0.26 (−0.13–0.65 Crl). Thus, they should not be considered as a viable option to slow down myopia progression.

#### 5.2.1. Soft Bifocal Contact Lenses

Several studies focused on the potential role of soft contact lenses (SCLs) in the reduction of myopia progression [62,87,88,89,90,91]. The most accredited theories explaining the rationale of use of SCLs would suggest a reduction of the hyperopic defocus and an imposition of a myopic defocus [87,92,93].

In this regard, Sankaridurg et al. showed in a 12-month non-randomized clinical study on Chinese children (baseline age 11.2 years old) how SCLs, designed to reduce the degree of relative peripheral hyperopia, decreased myopia progression of 34% (−0.57 D, 95% confidence interval [CI], −0.45–0.69 D), compared with spectacle lenses (−0.86, 95% CI, −0.74 to −0.99 D). However, despite the encouraging results, the study has some limitations. Firstly, since the mean age of treatment group was 11.6 years and the control group was 10.8 years, this age difference could have influenced the slower myopia progression in the treated group. Secondly, the control group should have been using ordinary contact lenses, instead of spectacles [93].

On the other hand, daily contact lenses with regular replacement may allow a better compliance for the children, compared to spectacles lenses and atropine [93,94] and a reduction in the risk of microbial keratitis due to overnight orthokeratology [95].

The CONTROL study recruited 86 multiethnic children aged 8 to 18 years old in a prospective, randomized clinical trial evaluating the effect of bifocal SCLs compared with the single vision contact lenses group (SVSCLs) on myopia progression over a period of 12 months. Children assigned to wear bifocal SCLs showed a pronounced relative reduction of 72% and 79%, respectively, in spherical equivalent and axial length (−0.79 ± 0.43 D compared with −0.22 ± 0.34 D of SVSCLs and 0.24 ± 0.17 mm compared with 0.05 ± 0.14 mm of SVSCLs control group) [61]. However, important limitations of this study are the short-term follow-up, (only 1 year) and the selection bias represented by the only inclusion of subjects with eso fixation disparity. Nonetheless, commercial interests should not influence the results of a study (since the first author had the patent of the lenses) and therefore it could be suggested that an independent study group should have carried out the study.

Another randomized, double-masked 2-year study, the Defocus Incorporated Soft Contact (DISC) trial, involved 221 children aged 8 to 13 in Hong Kong. DISC lenses encompassed concentric rings, providing an addition of +2.50 D and combining with the normal distance correction. The progression of the disease resulted 25% slower in children wearing DISC lenses than the SVSCLs group (0.30 D/year; 95% CI −0.71 to −0.47 vs. 0.4 D/year; 95% CI −0.93 to −0.65, *p* = 0.031). Moreover, a stronger reduction of 46% of myopia progression was found in relation to a higher time spent wearing DISC lenses (5 h or more/day).

The study showed in addition some limitations: a high dropout rate (42%) was found, predominantly occurred during the first year due to a lack of patients’ compliance; in addition, the retinal curvature profile examination was not performed, which could have altered the evaluation of the myopic defocus [62].

Moreover, the CLAMP Study and another randomized clinical trial published by Katz investigated the role of rigid gas permeable contact lenses (RGPCLs) in the control of myopia progression. However, both of the studies showed the absence of clinical efficacy in children wearing RGPCLs and therefore they should not be considered as a viable option to slow down myopia progression [96,97].

Recently, Cheng et al. evaluated the clinical effect of SCLs with positive spherical aberration (+SA) in proportion to conventional SCLSs in a sample of children aged 8 to 11. They divided the study into 2 phases: the first one designed for the treatment (1–2 years) and the second withdrawal phase including a 1.5-year follow-up. Although they found a significant reduction of axial elongation (AL) in the group wearing SCSLs with + SA at months 6 and 12 (respectively, 65.3% and 38.6% less, *p* < 0.05), and likewise a slower myopia progression of 0.21 D in the same cohort group at months 6 (54.0%, *p* = 0.05), no significant difference was found between the group neither in AL nor in SER during the withdrawal phase [92]. This would imply a negligible clinical effect of SCLs with +SA in the medium-long term.

The recent network meta-analysis published Huang et al. demonstrated that conventional SCLs compared to placebo or SVSLs had no overall significant effect on the control of myopia progression [−0.09, (−0.29–0.10 CrI)]. However, peripheral defocus modifying defocus SCLs resulted more effective than peripheral defocus modifying spectacles lenses, and more randomized clinical trials (RCTs) are needed to prove their efficacy [8].

#### 5.2.2. Orthokeratology

Orthokeratology (OrthoK) is a technique first experimented in the 1960s, involving the use of gas permeable contact lenses worn at night, which reshape temporally the corneal surface through a reverse geometry design. More specifically, OrthoK lenses work flattening the central cornea, which becomes thinner, redistributing the epithelial cells to the mid-periphery of the cornea. Thus, orthoK would slow myopia progression trough a reduction of the hyperopic peripheral refractive error and a resulting decrease in AL elongation [98,99].

The 2-year Longitudinal Orthokeratology Research in Children (LORIC) reported a reduction of 2.09 ± 1.34 D in the OrthoK group compared with the control group wearing SVSLs after 24 months. Additionally, the OrthoK group showed an AL elongation of 0.29 ± 0.27 mm, while the control group progressed to 0.54 ± 0.27 mm after the follow-up period [63]. Similar results were reported by the Retardation of Myopia in Orthokeratology (ROMIO) Study, a 2-year follow-up RCT comparing AL elongation in children aged 6 to 10 assigned to wear either OrthoK lenses or SVSLs. They revealed an average AL elongation of 0.36 ± 0.24 and 0.63 ± 0.26 mm in the OrthoK and SVSLs groups, respectively. Hence, patients wearing OrthoK lenses showed a slower overall reduction in AL elongation by 43%, compared with subjects wearing SVSLs. In particular, OrthoK lenses resulted more efficacious in younger myopic children (aged 7–8), with a faster myopic progression rate, causing a decrease in AL elongation from 65% in the control group to 20% in the OrthoK group [64]. The dropout rate in the study was 27% in the OrthoK group and 20% in the control group, whereas other studies reported a dropout rate in patients wearing OrthoK lenses included between 6 and 30%. Thus, these evidences would suggest a reduced compliance in children wearing OrthoK lenses, with almost one third of the patients interrupting the treatment after few months [100,101].

A recent meta-analysis included seven studies (two RCTs and five NRCTs) and found a −0.26 mm (95% CrI, −0.31 to −0.21) weighted mean difference for AL between the OrthoK group and the control group in a 2-year period follow-up [102]. Although a certain extent of OrthoK efficacy was shown, there are several limitations in this study; first of all, the number of the studies and the sample of patients analyzed were limited, secondly, the great variability between the targeted population and the study protocols could have restricted the reliability of the meta-analysis. Consequently, further larger-scale, randomized trials are needed in order to improve our knowledge with a long-term follow-up period and to better evaluate the real OrthoK efficacy.

In this perspective, the recent retrospective cohort study published by Lee et al. analyzed the association between overnight OrthoK and myopia progression over a period of 12-year follow-up. The OrthoK group had a significantly (*p* < 0.001) lower refractive error change compared with the control group during the follow-up period. Furthermore, the initial astigmatism power was identified as an important predictive variable for changes in refractive error. In fact, the presence of an astigmatism greater than or equal to −1.5 D, due to modifications in the corneal curvature, was more significantly associated with increased changes in refractive error over the follow-up years [103].

Although also Huang et al. showed an overall mild efficacy of OrthoK in comparison with placebo or SVSLs (−0.15 mm/year decrease in AL elongation, −0.22 to −0.08 CrI) [8], most of the studies lacked of a long-term wash-out period and did not focus thoroughly on the risk of myopia rebound. On this purpose, several studies reported that OrthoK discontinuation would lead to a faster myopia progression, comparable to children wearing SVSLs [104,105]. Yang et al. revealed in a retrospective study that the corneal morphology and central corneal thickness (CCT) returned to the original values after only 3 months of OrthoK discontinuation, however no effect of myopia rebound was shown [106].

Furthermore, several studies have highlighted an increased risk of infective keratitis secondary to OrthoK treatment and this evidence should not be neglected [107,108,109,110,111,112]. The prolonged overnight wear of OrthoK lenses is an important risk factor facilitating the spread of infections [113,114]. In a retrospective study, the incidence of infectious keratitis in children wearing overnight OrthoK lenses was 13.9 per 10,000 wearers every year [115]. A recent meta-analysis published by Kam et al. described the association between OrthoK lens wear and infectious keratitis, analyzing microbiological cultures. Remarkably, they found an overall positivity of microbiological cultures in 69.7% of the eyes studied (120/173 eyes), with Pseudomonas aeruginosa (36.4%) and Acanthamoeba (32.4%) as most commonly identified microorganisms. In addition, most of the patients with a positive microbiological culture resulted in the formation of corneal scars and 10% of them underwent surgical treatment [116]. Another retrospective study showed a higher percentage of patients (20.2%) who needed corneal transplantation, either elective or emergency, due to a perforation caused by contact lenses-associated infectious keratitis [117].

In conclusion, the adoption of OrthoK lenses to slow myopia progression in children should be carefully pondered, since the risk-benefit ratio combined with the low compliance of the patients has not given unidirectional results yet.

### 5.3. Pharmacological Treatments

The Cochrane database review in 2011 examined several drugs against myopia and assessed that, among them, antimuscarinic agents and in particular atropine would represent the most effective treatment to slow the progression of the disease [118]. The exact mechanism by which atropine, a non-selective muscarinic antagonist, would exert its effect should still be better clarified, but some studies would rule out a mechanism acting on the accommodative pathway [119,120] Atropine would rather be involved in a neurochemical cascade starting from the retina, as most of the evidences would suggest [121,122]. In this regard, Arumugam et al. showed in mammalian models that highly selective antimuscarinic agents such as MT7 (M1 receptors antagonist) and MT3 (M4 receptors antagonist) prevented form-deprivation myopia, acting on retinal muscarinic receptors at concentrations closer to the receptor affinity constants rather than concentrations found in sclera and choroid [123,124]. Furthermore, it was demonstrated an increased release of dopamine by the RPE following atropine injection both in vivo and in in vitro studies [125].

Also, the role of ocular hypotensive drugs (β-adrenergic blockers) has been investigated in some clinical trials, because the increased intraocular pressure was hypothesized to cause a passive stretching of the sclera with a following growth of the eye size [126]. A randomized clinical study compared the efficacy of a β-blocker drug, timolol 0.25%, with spectacles in 150 Danish children. However, after a 2-year follow-up period, they found no significant differences in the myopia progression rate between the two groups (−0.59 D per year in the timolol group compared with −0.57 D per year in the single vision group) [121]. In this regard, the recent meta-analysis published by Huang et al. showed that timolol was inferior to most of the interventions, with an overall treatment effect even smaller than SVSLs or placebo (−0.02 D, −0.29 to 0.10) [8].

Given the findings in animal models and its potential role in reinforcing the posterior sclera and in preventing axial elongation, 7-methylxanthine (7-mx), an adenosine antagonist, has been investigated in the prevention of myopia [40,127]. Moreover, in primates 7-mx has been proven to reduce the axial myopia produced experimentally by hyperopic defocus [128]. In humans, a randomized, 36-month pilot study evaluated the effect of systemic administration of 7-mx in 68 myopic children and revealed its efficacy in decreasing axial myopic elongation in comparison with placebo after 24 months of treatment. In the third year of follow-up, after the 7-mx treatment was discontinued, both of the groups were aligned again with increase in axial elongation, proving the efficacy of the adenosine agonist [41]. However, no further studies on humans were carried out and therefore it is difficult to draw any reliable conclusion on the clinical efficacy of this drug. Thus, further studies are needed to better investigate its potential role in the prevention of myopia.

Furthermore, also the potential role of latanoprost, a prostaglandin analog, is being investigated in animal models. In fact, as previously explained, the rationale of the use of hypotensive drugs would reside in the lower pressure exerted on the sclera and subsequently a decreased axial elongation [126]. In a recent study, latanoprost has shown to be effective in reducing not only the intraocular pressure, but also axial elongation and refractive error in guinea pigs [129]. Thus, given the encouraging results of this drug commonly used in the treatment of glaucoma, further studies should better define its possible role in the prevention of myopia, not neglecting the tolerability and the possible side effects of a hypotensive drug in children.

Tropicamide, a short acting cycloplegic drug, showed in a study including 25 twins in the United States treated with a combination of 1% tropicamide eye drops and BSLs no efficacy in myopia progression after 3½ years of follow up [130]. Hence, due to the short duration of action combined with the poor clinical efficacy further studies on tropicamide have not been conducted.

Pirenzepine, a selective M1 receptors antagonist, has been investigated in several studies because is less likely to cause atropine-related side effects like cycloplegia and mydriasis. Animal models have shown efficacy of this antimuscarinic agent in slowing experimental-induced myopia progression [131,132].

The Asian Pirenzepine Study Group evaluated in a double-masked, multicenter study the safety and efficacy of pirenzepine 2% ophthalmic gel applied twice a day in children aged 6–12. After one year of treatment the pirenzepine group showed better outcomes in comparison with the placebo group in terms of refractive status and axial length (0.47 D vs. 0.84 D, *p* < 0.001) and (0.20 mm vs. 0.33 mm, *p* = 0.008) respectively, with a tolerable dropout rate (11%) due to adverse events [65]. Analogously the 2-year randomized US Pirenzepine Study Group reported in children aged 8 to 12 a slower myopia progression in the pirenzepine group compared with the placebo one (0.58 D vs. 0.99 D, *p* = 0.008). Moreover, pirenzepine ophthalmic gel 2% provided a good safety profile; in fact, the most frequent adverse events, such as papillae/follicles, medication residue and abnormalities of accommodation, resulted mild to moderate in severity and the dropout rate was only 11% of the patients in the 2-year follow-up [66]. Although both of the studies showed promising results in slowing myopia progression with a good compliance of the patients, further clinical trials investigating pirenzepine were not performed, probably overshadowed by the clinical relevance of atropine in ATOM 1 and ATOM 2 trials [67,133]. However, laboratory research in vitro and vivo on this drug is still continuing and, in a recent study published in 2017, pirenzepine was encapsulated into micelles with acid sorbic and displayed an improvement of its bioa-vailabilty and corneal permeability thanks to the above-mentioned lipophilization process [134]. In the near future, given the encouraging laboratory and clinical evidence, further RCT are needed to better describe the application of pirenzepine in slowing myopia progression.

Among the antimuscarinic drugs, also cyclopentolate has shown promising results in the treatment of myopia progression. In fact, Huang et al reported an overall moderate effect in comparison with SVSLs or placebo, with an overall gain of 0.33 D (−0.22 to 0.67) based on network meta-analysis [8]. However, in 1989 a randomized clinical trial had already reported a clinical efficacy inferior to atropine. In the study they randomized patients into 3 groups: the first group was treated with atropine 1% eye drops at night, the second with cyclopentolate 1% eye drops at night and the third received placebo (normal saline) eye drops. After 1 year of treatment, the myopic progression rate was −0.219 D in the atropine group, −0.578 D in the cyclopentolate group, and −0.914 D in the saline group [135]. These results would suggest a moderate effect displayed by cyclopentolate in the management of myopia, but inferior in efficacy than atropine.

In 1984 Brodstein et al. had already pointed out the role of atropine 1% in slowing myopia progression in a sample of 253 American children followed up for up to 9 years [136]; however, the non-randomized nature of the study does not allow to draw significant conclusions from these results.

The Atropine in the Treatment of Childhood Myopia study (ATOM 1) was designed as a randomized, placebo-controlled, double-masked trial and recruited 400 Asian children aged 6 to 12 years with refractive error of SED between −1.00 D and −6.00. Children were randomized to receive either atropine 1% or placebo vehicle drops unilaterally in one eye at bedtime and changes in SED and AL were measured by cycloplegic autorefraction and ultrasonography. After a 2-year treatment period, 86.5% of the patients completed the study and the atropine-treated group showed a significant slower myopia progression with a difference of −0.92 D in SED (95% confidence interval, −1.10 to −0.77 D; *p* < 0.001) and 0.40 mm in AL (95% confidence interval, 0.35–0.45 mm; *p* < 0.001) compared with the placebo. In addition, no serious adverse effect was described in the treated population, except for allergic reactions (4.5%), glare (1.5%) and blurred near vision (1%) [67].

In 2009 Tong et al. analyzed the myopia progression in the same population of ATOM 1 after a 1-year washout period. Overall, after 3 years (including 2 years of treatment and 1 year of washout period) children treated with atropine 1% showed a less severe myopia progression (-4.29 ± 1.67 D vs. −5.22 ± 1.38 D, *p* > 0.001). However, during the washout period there was a significant higher rate of myopia progression in the atropine-treated group (−1.14 ± 0.80 D) compared with the placebo group (−0.38 ± 0.39 D, *p* < 0.0001). These results revealed that atropine 1% discontinuation led to a rebound phenomenon, especially in the first 6 months of the washout period. In this regard, the authors would identify the cycloplegic effects of atropine as the underlying cause of the rebound phenomenon, because they found changes in accommodation but not in AL [137].

The Atropine 2 Treatment of Childhood Myopia (ATOM 2) study aimed to compare the clinical efficacy and safety of lower doses of atropine with ATOM1 historical controls. 400 Asian children aged 6 to 12 years with myopia of at least −2.0 D were enrolled and randomized to be treated in a 2:2:1 ratio with atropine 0.5%, atropine 0.1% and atropine 0.01%, respectively. After a 2-year treatment period, the mean myopia progression was −0.30 ± 0.60, −0.38 ± 0.60, and −0.49 ± 0.63 D in the atropine 0.5%, 0.1%, and 0.01% groups, respectively (*p* = 0.02 between the 0.01% and 0.5% groups; between other concentrations *p* > 0.05). By comparison, the myopia progression in ATOM1 study resulted −1.20 ± 0.69 D in the placebo group and −0.28 ± 0.92 D in the atropine 1%-treated group. Atropine 0.01%, even if initially assumed to have a minimal effect on myopia progression and therefore used as a potential control, displayed a significant clinical effect (−0.49 D ± 0.63 D/2 years) with smaller differences in SED and AL with the higher-dose atropine 0.5% group (0.19 D and 0.13 mm/2 years, respectively). In addition, atropine 0.01% provided a better ocular side effect profile than higher dose groups, with the accommodation remaining at 11.8 D (compared with 6.8 D and 4 D in the 0.1% and 0.5% atropine groups, respectively) and a mean photopic pupil size change after 2 years of 0.74 mm (2.25 mm and 3.11 mm in the 0.1% and 0.5% groups, respectively) [133].

Furthermore, Chia et al. extended the ATOM 2 study evaluating myopia progression after 1 year of washout period. Like the previous ATOM 1 study, they highlighted a dose-related rebound phenomenon in the 89% of the patients who completed the washout period. In fact, children treated with atropine 0.5% showed a faster myopia progression (−0.87 ± 0.52 D), compared with the 0.1% group (−0.68 ± 0.45 D) and the 0.01% group (−0.28 ± 0.33 D, *p* < 0.001). AL elongation was also more significant in the 0.5% (0.35 ± 0.20 mm) and 0.1% (0.33 ± 0.18 mm) eyes, in comparison with the 0.01% eyes (0.19 ± 0.13 mm, *p* < 0.001) [138].

In addition, they selected children originally assigned to the 0.5%, 0.1%, 0.01% groups in ATOM2 with a myopia progression rate higher than -0,5 D after the washout period and retreated them with atropine 0.01% along an additional 2-year treatment period. They reported an overall myopia progression of −1.38 ± 0.98 D, −1.83 ± 1.16 D, −1.98 ± 1.1 D in the 0.01%, 0.1%, 0.5% groups, respectively [139]. In conclusion, atropine 0.01% drops not only caused the least rebound effect after the washout period, but also guaranteed over the long-term period the best clinical efficacy in slowing down myopia progression and the most tolerable side effects in comparison with higher doses.

However, although ATOM 1 and ATOM 2 have shown promising results in the management of myopia progression, there is an important selection bias in the population, since only children of Asian ethnicity have been investigated in these studies. For this reason, Polling et al. evaluated the efficacy of atropine 0.5% in a sample of 77 children in the Netherlands with high myopia (−6.6 D mean SER at baseline), composed by children of European (n = 53), Asian (n = 18) and African (n = 6) descents. The 78% of the children completed the 1-year treatment period, showing a significant reduction in myopia progression rate, decreasing from (−1.0 D/year ± 0.7) before the treatment to (−0.1 D/year ± 0.7) after the 12 months. Despite the high incidence of dose-related side effects like photophopia (78%), reading problems (38%), headaches (22%), atropine showed an important clinical efficacy in slowing down progressive myopia also in European children. However, given the non-randomized nature of the study, conclusions should be drawn very carefully from it [140].

Similarly, Clark et al. reported in a retrospective case-control study including 60 American children the significant decrease in myopia progression after 1-year atropine 0.01% treatment (−0.1 ± 0.6 D/year in the treated group vs. −0.6 ± 0.4 D/year in the control group) [141].

Loughman et al. studied the acceptability of atropine 0.01% in a population of Caucasian students aged 18–27. Although they found a statistical modification of pupil size (*p* = 0.04) and responsiveness (*p* < 0.01), the visual acuity and the reading speed were not significantly affected and therefore they confirmed an overall good impact on the patients’ quality of life [142].

Recently, based on the results of ATOM 1 and ATOM 2 studies, Schittkoswki et al. published practical guidelines dealing with the posology of atropine 0.01% in the treatment of myopia. In these guidelines atropine 0.01% treatment should last 2 years; in fact, as already reported by Chia et al., atropine would exert an increased efficacy during the second year of treatment [139]. After the 2-year period, the administration of atropine should be stopped if the myopia progression rate is slower than 0.25 D/year during the second year; on the contrary, a myopia progression rate faster than 0.5 D/year would justify a resumption of the treatment. Moreover, these guidelines would suggest a 6-month follow-up, including cycloplegic refraction and axial length measurements [143].

Hence, most of the evidences seem to agree with the administration of atropine 0.01% as a first line treatment in the prevention of myopia progression; however, a recent prospective cohort Indian study published in 2017, aimed to evaluate the clinical efficacy of atropine 1% in children with progressive myopia (defined as ≥ −0.5 D/year) and high myopia at baseline (mean baseline sphere −5.2 D). After a mean 23-month follow-up the disease progression rate decreased from −0.6 D/year (range −0.5 D/year to −3 D/year) to −0.2 D/year (range 0 D/year to −1.5 D/year) after atropine 1% daily application. For this reason, the authors would suggest a potential role of atropine 1% as a second line treatment for rapid progressions and non-responders to lower doses of atropine; however, an important limitation of the study is that the washout period and the rebound effect were not evaluated and, therefore, further studies should provide a long-term efficacy and safety profile of atropine 1% [144]. In addition, the population analyzed was over 90% ethnic Chinese, and therefore further clinical trials in other ethnic populations should be performed.

In the network meta-analysis Huang et al. reported that the treatment effect of atropine (high, moderate and low-dose) in comparison with placebo or SVLSs was strong and in particular high-dose atropine (1% and 0.5%) was significantly superior (*p* < 0.05) to other interventions, except for moderate-dose atropine (0.1%) and low-dose atropine (0.01%) [8].

In conclusion, although atropine has shown a dose-related clinical efficacy in slowing down myopia progression, the low-dose formulation could be considered the best candidate treatment because of the minimal clinical side effects and the least rebound phenomenon (Table 2).

## 6. Discussion

Myopia has been recognized worldwide as one of the most compelling ocular diseases by the World Health Organization’s Global Initiative for the Elimination of Avoidable Blindness [153]. Moreover, the Gutenberg Eye Study reported in the UK a comparable prevalence of the disease between Asian students (53.4%) and Caucasian students (50%), underlying the growing epidemic spread also in the Western countries [154].

Thus, many prophylactic interventions have been investigated in order to prevent or at least to slow down the progression of the disease.

Several studies have demonstrated that the genetic background plays an important role in the development of myopia; in fact, a positive association between parental myopia and child’s risk of developing the condition has been shown [155]. However, genetics represents obviously a non- modifiable variable and, for this reason, prevention should start from simple behavioral strategies dealing with the reduction of environmental risk factors. From the recent meta-analysis published by Xiong et al. prolonged outdoor activities would be more significantly involved in the reduction of myopia onset, rather than its progression [52]. However, although the increase of outdoor activities would exert an overall modest effect on myopia progression, they should be considered more comprehensively as a simple and far-sighted strategy aiming to improve the global health of the child [49,50,51]. In fact, several studies reported the positive association between outdoor physical activities and a good health condition in children [156,157,158].

The clinical efficacy of both PALs and BSLs resulted to be small in several clinical trials and therefore they should not be considered as a first line treatment in the prevention of myopia progression [68].

The CONTROL and the DISC Studies reported in children wearing SCLs a reduction of myopia progression by 72% after a 1-year period follow-up and by 25–46% after 24 months, respectively [61,62]. However, the high dropout rate reported (42%), the additional skills required by contact lenses wearers and the lack of longer-term efficacy evidence represent an important limitation in the adoption of SCLS in children to slow myopia progression.

On the other hand, orthoK lenses have shown the most relevant effect on the AL elongation reduction in several RCTs. The LORIC Study reported a decrease of AL elongation by 46% in orthoK lenses compared to SVLSs after a 24-month follow-up and in parallel the ROMIO Study revealed an overall reduction in AL elongation by 43%, especially in younger children with faster myopia progression rate [63,64]. However, further clinical trials are needed to provide a better knowledge of changes in SER and more evidences along a longer-term follow-up with a thorough evaluation of the washout period. In fact, some studies reported the likely presence of rebound phenomenon after ortho-K lenses discontinuation, with a faster myopia progression rate revealed mainly in the first 3 months [106]. In addition, several studies revealed a low compliance of the patients wearing ortho-K lenses, mostly due to a lack of motivation for wearing everyday overnight contact lenses [100,101]. Nonetheless, the significant risk of developing infectious keratitis in overnight ortho-K wearers constitutes an important factor that should not be neglected, exposing the children to possibility of sight-threatening complications [112,116]. Ultimately, weighing the risk-benefit ratio, ortho-K lenses should not be considered the first choice in the prevention of myopia progression.

From ATOM 1 and ATOM 2 Trials emerged the dose-related clinical efficacy of atropine, from an overall 50% myopia progression reduction with atropine 0.01% to a 77% decrease with atropine 1%. However, higher doses of atropine have shown a more significant rebound phenomenon and a higher incidence of ocular side effects like allergic reactions, glare and blurred near vision in comparison with atropine 0.01% [67,133]. In this regard, the latter formulation demonstrated the most tolerable safety profile for the children combined with the least rebound phenomenon after discontinuation, leading to most convincing long-term results in order to be considered the first line treatment in slowing myopia progression [73,146,150]. However, further studies are needed to provide globally broad range results in other populations and give more appropriate instructions on the posology of the drug.

In addition, also pirenzepine 2% ophthalmic gel has shown encouraging results in children, however only 2 RCTs have been reported and larger-scale trials are needed to better evaluate its role in slowing myopia progression [65,66].

## 7. Conclusions

In conclusion, antimuscarinic drugs and in particular atropine 0.01% have reported the best evidences on clinical efficacy combined with negligible side effects, a good tolerability and compliance for the patients. However, the possible myopic rebound after the suspension of the treatment and the convenience in using an eye drops daily for years to obtain a reduction of the myopia progression of moderate clinical impact may be questionable. Furthermore, it would be interesting analyzing the potential additive effects of antimuscarinic drugs combined with other therapies in larger-scale RCTs.

## Figures and Tables

**Table 1:** Randomized clinical trials investigating the clinical efficacy of each interventions in slowing myopia progression.

Author(s), Year	Study Design	Intervention Investigated	Total No, Ethnicity and Age Range	Baseline SER and/or AL	Final Follow-up	Results
Aller TA, 2016 [61]	Randomized	SCLs	186, American, 8–18	−2.69 ± 1.40 D	1 year	Control group: −0.79 ± 0.43 D progression Treated group: −0.22 ± 0.34 D
Lam CS, 2014 [62]	Randomized, double-blind	SCLs	221, Hong Kong, 8–13	−1.00 to −5.00 D	2 years	Control Group: 0.40 D/year and 0.18 mm/year Treated Group: 0.30 D/year and 0.13 mm/year
Cho P, 2005 [63]	Pilot study	OrthoK lenses	35, Hong Kong, 7–12		2 years	Gain of 2.09 ± 1.34 D for the treated group
Cho P, 2012 [64]	Randomized, single-masked	Ortho-K lenses	102, Hong Kong, 6–10	0.50 to 4.00 D	2 years	Control group: 0.63 ± 0.26 mm AL elongation Treated Group: 0.36 ± 0.24 mm
Tan DT, 2005 [65]	Randomized, placebo-controlled, double-masked	Pir 2% ophthalmic gel	353, Singapore, 6–12	−0.75 to 4.00 D	1 year	Control group: 0.84 D myopia progression Pir/PBO group = 0.70 D Pir/Pir group = 0.47 D
Siatkowski RM, 2008 [66]	Randomized, placebo controlled, double-masked	Pir 2% ophthalmic gel	174, American, 8–12	−0.75 to −4.00 D astigmatism </= 1.00 D	2 years	0.41 D gain for the treated group
Chua WH, 2006 (ATOM 1) [67]	Randomized, placebo-controlled, double masked	Atr 1%	400, Asian, 6–12	1.00 to −6.00 D	2 years	Control group: −1.20 ± 0.69 D and 0.38 ± 0.38 mm myopia progression Treated group: 0.28 ± 0.92 D and −0.02 ± 0.35 mm
The Comet Group, 2001 [68]	Randomized	PALs	469, American,9	Between −1.25 and −4.50 D	3 years	Treated group gain of 0.2 D

AL = axial elongation Atr = atropine; BSLs = bifocal spectacle lenses; D = diopters; Ortho-K = orthokeratology; PASL = progressive addition spectacles lenses; PBO = placebo; PBSLs = prismatic bifocal spectacles lenses; Pir = pirenzepine; SCLs = soft contact lenses SER= spherical equivalent refraction.

**Table 2:** Treatment efficacy of each intervention compared with Single Vision Spectacles Lenses/Placebo (based on Huang et al. network meta-analysis) [8].

Intervention	Mechanism of Action	Overall Gain in SER and/or Decreased AL Elongation	Degree of Efficacy (0–3)
Atr H	Putative non-accomodative pathway, increase retinal DA levels [126,145]	SER: 0.68 (0.52–0.84) AL: −0.21 (−0.28 to −0.16)	3
Atr L	Putative non-accomodative pathway, increase retinal DA levels	SER: 0.53 (0.21–0.85) AL: −0.15 (−0.25 to −0.05)	3
Pir	Selective M1 receptor antagonist, same putative mechanism of atropine	SER: 0.29 D (0.05 to 0.52) AL: −0.09 (−0.17 to −0.01)	2
Cyc	Antimuscarinic agent, same putative mechanism of atropine	SER: 0.33 (−0.02 to 0.67)	2
Ortho-K lenses	Reduction of the hyperopic peripheral refractive error, reshaping the temporal surface of cornea [146]	AL: −0.15 (−0.22 to −0.08)	2
PDMCLs	Reduction of peripheral myopic defocus	SER: 0.21 (−0.07 to 0.48) AL: −0.11 (−0.20 to −0.03)	2
PASLs	Putative reduction of the retinal hyperopic blur by decreasing the accommodative lag during near work [83]	SER: 0.14 (0.02 to 0.026)	1
BSLs	Putative reduction of the retinal hyperopic blur by decreasing the accommodative lag during near work [147]	SER: 0.09 (−0.07 to 0.25) AL: −0.06 (−0.12 to 0.00)	1
SCLs	Putative reduction of peripheral hyperopia [148]	SER: −0.09 (−0.29 to 0.10)	1
MOA	Putative increased release of DA induced by light exposure [149]	SER: 0.14 (−0.17 to 0.46)	1
Tim	Reduction of intraocular pressure would reduce scleral stretching [150]	SER: −0-02 (−0.31 to 0.27)	0
RGPCLSs	Putative reduction of peripheral hyperopia [151]	AL: 0.02 (−0.05 to 0.10)	0
USVSLs	Spectacles designed to reduce the peripheral hyperopic defocus	SER: −0.11 (−0.35 to 0.13) AL: 0.03 (−0.06 to 0.11)	0
Trop	Cycloplegic agent, same putative mechanism of atr [152]	No randomized studies	0
Biofeedback visual training	Modification of autonomic nervous system in order to help the accommodation process [69]	No randomized studies	0

AL = axial elongation Atr = atropine; Atr H = high-dose atropine (1%, 0.5%); Atr L = low-dose atropine (0.01%) BSLs = bifocal spectacle lenses; Cyc = cyclopentolate; D = diopters; MOA = more outdoor activities (14–15 h/week) OrthoK = orthokeratology; PASLs = progressive addition spectacles lenses; PBSLs = prismatic bifocal spectacles lenses; PDMCLSs = peripheral defocus modifying contact lenses; Pir = pirenzepine; RGPCLs = rapid gas-permeable contact lenses; SCLs = soft contact lenses SER= spherical equivalent refraction; Tim = timolol; Trop = tropicamide; USVSLs = undercorrected single vision spectacle lenses. Degree of efficacy: 3 = most effective; 2 = moderately effective; 1 = less effective; 0 = ineffective.
